# Clinical Implications of Estimating Glomerular Filtration Rate with Three Different Equations among Older People. Preliminary Results of the Project “Screening for Chronic Kidney Disease among Older People across Europe (SCOPE)”

**DOI:** 10.3390/jcm9020294

**Published:** 2020-01-21

**Authors:** Andrea Corsonello, Regina Roller-Wirnsberger, Gerhard Wirnsberger, Johan Ärnlöv, Axel C. Carlsson, Lisanne Tap, Francesco Mattace-Raso, Francesc Formiga, Rafael Moreno-Gonzalez, Christian Weingart, Cornel Sieber, Tomasz Kostka, Agnieszka Guligowska, Pedro Gil, Sara Lainez Martinez, Rada Artzi-Medvedik, Itshak Melzer, Fabrizia Lattanzio

**Affiliations:** 1 Italian National Research Center on Aging (IRCCS INRCA), 60124 Ancona, Italy; a.corsonello@inrca.it (A.C.); f.lattanzio@inrca.it (F.L.); 2 Department of Internal Medicine, Medical University of Graz, 8036 Graz, Austria; gerhard.wirnsberger@medunigraz.at; 3 Department of Medical Sciences, Uppsala University, 752 36 Uppsala, Sweden; johan.arnlov@ki.se (J.Ä.); axel.carlsson@ki.se (A.C.C.); 4 School of Health and Social Studies, Dalarna University, 791 31 Falun, Sweden; 5 Division of Family Medicine and Primary Care, Department of Neurobiology, Care Science and Society, Karolinska Institutet, 141 52 Huddinge, Sweden; 6 Section of Geriatric Medicine, Department of Internal Medicine, Erasmus MC, University Medical Center Rotterdam, 3015 GD Rotterdam, The Netherlands; l.tap@erasmusmc.nl (L.T.); f.mattaceraso@erasmusmc.nl (F.M.-R.); 7 Geriatric Unit, Internal Medicine Department, Bellvitge University Hospital–IDIBELL, 08907 L’Hospitalet de Llobregat, Barcelona, Spain; fformiga@bellvitgehospital.cat (F.F.); rmorenog@bellvitgehospital.cat (R.M.-G.); 8 Department of General Internal Medicine and Geriatrics, Institute for Biomedicine of Aging, Krankenhaus Barmherzige Brüder, Friedrich-Alexander-Universität Erlangen-Nürnberg, 93049 Regensburg, Germany; Christian.Weingart@barmherzige-regensburg.de; 9 Department of Internal Medicine-Geriatrics, Institute for Biomedicine of Aging (IBA), Friedrich-Alexander Universität Erlangen-Nürnberg, Koberger Strasse 60, 90408 Nuremberg, Germany; cornel.sieber@fau.de; 10 Department of Geriatrics, Healthy Ageing Research Centre, Medical University of Lodz, 90-647 Lodz, Poland; tomasz.kostka@umed.lodz.pl (T.K.); agnieszka.guligowska@umed.lodz.pl (A.G.); 11 Department of Geriatric Medicine, Hospital Clinico San Carlos, 28040 Madrid, Spain; pgil@salud.madrid.org (P.G.); slainezm@outlook.es (S.L.M.); 12 The Recanati School for Community Health Professions at the faculty of Health Sciences, Ben-Gurion University of the Negev, 84105 Be’er Sheva, Israel; rada.artzi@gmail.com (R.A.-M.); itzikm@bgu.ac.il (I.M.)

**Keywords:** chronic kidney disease (CKD), Berlin Initiative Study (BIS), Full Age Spectrum (FAS), estimated glomerular filtration rate (eGFR), older patients, sarcopenia, muscle mass, sex

## Abstract

We aimed at investigating to what extent CKD may be staged interchangeably by three different eGFR equations in older people, and evaluating the source of discrepancies among equations in a population of 2257 patients older than 75 years enrolled in a multicenter observational study. eGFR was calculated by CKD-EPI, BIS and FAS equations. Statistical analysis was carried out by Bland–Altman analysis. κ statistic was used to quantify the agreement between equations in classifying CKD stages. The impact of selected variables on the difference among equations was graphically explored. The average difference between BIS and FAS was −0.24 (95% limits of agreement (95%LA = −4.64–4.14) mL/min/1.73 m^2^. The difference between CKD-EPI and BIS and between CKD-EPI and FAS was 8.97 (95%LA = −2.90–20.84) and 8.72 (95%LA = −2.11–19.56) mL/min/1.73 m^2^, respectively. As regards CKD stage classification, κ value was 0.47 for both CKD-EPI vs. FAS and CKD-EPI vs. BIS, while BIS and FAS had similar classificatory properties (κ = 0.90). Muscle mass was found related to the difference between CKD-EPI and BIS (R^2^ = 0.11) or FAS (R^2^ = 0.14), but not to the difference between BIS and FAS. In conclusion, CKD-EPI and BIS/FAS equations are not interchangeable to assess eGFR among older people. Muscle mass may represent a relevant source of discrepancy among eGFR equations.

## 1. Introduction

Estimated glomerular filtration rate (eGFR) equations are routinely used for clinical assessment of kidney function, despite their accuracy among older patients still being a matter of debate. Identifying appropriate filtration markers and estimating equations for older and especially frail older people has come into focus and is of clinical as well as public interest as the prevalence of chronic kidney disease (CKD) is known to increase with age and to impact health status and survival in several different populations [1,2]. Timely detection of CKD allows to contrast some pathogenetic mechanisms such as uncontrolled hypertension or, in diabetic nephropathy, glomerular hyperfiltration in order to slow kidney function decline [3]. Importantly, it also allows to tailor the dosage of kidney-cleared medications, as well as CKD stage-specific interventions [4].

To address current inconsistencies across recently published studies on determination of kidney function in older patients, it seems necessary to consider different statistical approaches, laboratory assays used to measure creatinine and specimen collection, handling, and storage. Furthermore, the impact of parameters like muscle mass, may impact internal consistency of measurement of kidney function, especially in this cohort of older subjects [5]. Indeed, sarcopenia, which is commonly observed among frail older people, may reduce creatine production leading to low serum creatinine levels even despite a significantly reduced glomerular filtration rate (GFR) [6]. To this aim, several different eGFR equations have been developed and tested for these cohorts of patients [7,8,9,10,11]. Since 2012, KDIGO has adopted The Chronic Kidney Disease Epidemiological Collaborative (CKD-EPI) equation, but it cannot be considered universal in clinical practice yet [4]. This equation was developed from a population consisting of 8254 subjects pooled from 10 studies, including 13% of people aged >65 years and 28% diabetics, and externally validated in a population of 3896 subjects pooled from 16 other studies [8]. The Berlin Initiative Study (BIS) [9] has been developed to be used in elderly people, and Full Age Spectrum (FAS) equations for the whole life span adapting also for age and both equations have been externally validated against gold-standard measured GFR [12,13]. Several studies tried to compare the sensitivity of the different creatinine-based equations (CKD-EPI, BIS, FAS) in cohorts of older subjects [14] with striking differences in results. Nevertheless, creatinine-based eGFR is still the most widely used measure for clinical assessment of kidney function. Other biomarkers of kidney function, especially cystatin C, were investigated in an attempt to improve the accuracy of kidney function estimates. While the accuracy of equations including both creatinine and cystatin C in predicting measured GFR was found to be better than that observed with creatinine-based ones among older patients [15], the agreement between equations was found to be only marginally improved [16] and prognostic accuracy unchanged when adding cystatin C [17]. Thus, the additional costs generated by cystatin C assessment may not be associated with a true improvement in clinical assessment of kidney function. Indeed, it has been suggested that cystatin C may be cost-effective in young adults where it can help to reduce the number of false positives, but not in individuals aged ≥ 75 years [18]. Additionally, even the accuracy of cystatin C-based eGFR in predicting measured GFR was found to improve when including fat-free mass in kidney function assessment among older CKD patients [19]. It is therefore evident that a knowledge gap still exists and ongoing studies will likely help to bridge it [20]. Meanwhile, creatinine-based eGFR remains the less expensive and most widely available screening measure of kidney function.

Considering albumin-to-creatinine ratio (ACR) for staging of chronic kidney disease, the picture in ageing patients in daily clinical practice becomes even more complex [21]. Albuminuria and GFR are both relevant measures of the functionality of glomeruli. Albuminuria is mainly a measure of the glomerular capillary wall permeability to macro-molecules and increased albuminuria occurs earlier in the course of many kidney diseases compared to GFR decline [22]. Both parameters play an important role in detection and staging of CKD. The current evidence for the validity of these two surrogate markers for prediction and progression of CKD is stronger for GFR than for change of albuminuria over time [21]. However, during ageing the sensitivity of GFR determination and mathematical models applied to measure creatinine in the available test systems are strongly impacted by muscle mass. As early detection of a decline in kidney function is a key element in clinical complex care management for many doctors, the aim of the present study was to test how the mathematical models for eGFR calculation are affected by muscle mass and function as measured with bio-impedance analysis (BIA) and short physical performance battery (SPPB) [23], two simple tests applicable in daily clinical practice in a cohort of multi-morbid 75 years+ patients in different stages of CKD at time of inclusion. We also aimed at investigating how difference in eGFR between CKD-EPI, BIS and FAS formula may affect the predictive staging of patients when introducing ACR according to KDIGO guidelines [4].

## 2. Materials and Methods

The SCOPE study (grant agreement number 436849), is a multicenter 2-year prospective cohort study involving patients older than 75 years attending geriatric and nephrology outpatient services in participating institutions in Austria, Germany, Israel, Italy, the Netherlands, Poland and Spain. Methods of the SCOPE study have been extensively described elsewhere [20]. Patients were requested to sign a written informed consent before entering the study. The study protocol was approved by ethics committees at all participating institutions, and complies with the Declaration of Helsinki and Good Clinical Practice Guidelines. Only baseline data are used in the present study.

Overall, 2461 patients were initially enrolled in the study. Of them, 204 patients were excluded from this study because of incomplete baseline data, thus leaving a final sample of 2257 patients to be included in the analysis. For testing the hypothesis of impact of muscle mass on detection of glomerular filtration rate, a sub-cohort of 1462/2257 participants was recruited for muscle mass measurements as outlined below.

### 2.1. Study Variables

Serum creatinine was measured at local level by standard methods. Creatinine-based eGFR was calculated using the equations described in Table 1.

Albumin in urine was detected by urine spot analysis and expressed as mg albumin per gram urine (mg/g); ACR was calculated according to KDIGO guidelines [4].

Variables included in further analysis were age, sex and Body Mass Index (BMI) using the formula recommended in the guidelines of the European Society of Clinical Nutrition (ESPEN) [24].

Physical performance was included in the analysis for consideration of sarcopenia. Physical performance was measured by SPPB [25]. The SPPB includes gait speed (usual time to walk 4 m), five chair-stands test (time to rise from a chair and return to the seated position five times without using arms), and balance test (ability to stand with the feet together in the side-by-side, semi-tandem, and tandem positions). A score from 0 to 4 was assigned to performance on each task. Individuals received a score of 0 for each task they were unable to complete. Summing the three individual categorical scores, a summary performance score was created for each participant (range, 0–12), with higher scores indicating better lower body function.

To further validate muscle mass measures in comparison to SPPB values in a sub-cohort of 1462 participants in the SCOPE study, BIA was carried out by Akern BIA101 with BodyGram PLUS software (Akern srl, Pontassieve (FI), Italy), and muscle mass was calculated using the Janssen et al. equation [26]. BIA was not performed in patients with pacemaker or implantable cardioverter defibrillator.

### 2.2. Analytic Approach

Statistical analysis was performed by SPSS Statistical Software Package for Win V21.0 (SPSS Inc, Chicago, IL, USA) and MedCalc (MedCalc software bv, Ostend, Belgium). To investigate the impact of selected study variables on differences among equations, we used a graphic approach by GraphPad Prism 8 (GraphPad, San Diego, CA, USA).

Demographic and clinical characteristics of participants were expressed by descriptive statistics and the prevalence of selected disease was counted and expressed in percent of people affected in the cohort. Non-parametric tests were applied to calculate differences between groups.

Crude correlation among glomerular filtration rate calculated by CKD-EPI, BIS and FAS equation was investigated graphically. Bland–Altman plots were generated to plot the difference CKD-EPI-BIS, CKD-EPI-FAS and BIS-FAS against the mean of the two estimates, respectively, or the whole cohort of participants.

Furthermore, the prevalence of CKD stages obtained with different equations was investigated adding ACR and creatinine based glomerular filtration rates according to KDIGO guidelines [4]. Cohen’s kappa (κ) was calculated to quantify the agreement between equations in identifying people with different degrees of kidney dysfunction (eGFR > 90, stage 1; 90–60, stage 2; 60–45, stage 3a; 45–30, stage 3b; and <30 mL/min/1.73 m^2^, stage 4–5). Finally, we also calculated the prevalence of each individual KDIGO stage of CKD based on eGFR and ACR. Analyses were further stratified by sex.

Finally, to investigate the impact of sarcopenia on the observed difference among study equations, we used a graphic approach plotting the difference of the values obtained by two equations on the value of the variable of interest (BMI, SPPB or muscle mass) and using local regression techniques to fit a parametric or non-parametric curve smoothing the relationship between the two variables. We adapted our choice on the basis of the regression curve best fitting the given distribution to calculate regression analysis.

### 2.3. Ethical Statement

The study protocol was approved by ethics committees at all participating institutions, and complies with the Declaration of Helsinki and Good Clinical Practice Guidelines. Only baseline data are used in the present study. Ethics approvals have been obtained by Ethics Committees in participating institutions as follows:

- Italian National Research Center on Aging (INRCA), Italy, #2015 0522 IN, 27 January 2016.
- University of Lodz, Poland, #RNN/314/15/KE, 17 November 2015.
- Medizinische Universität Graz, Austria, #28–314 ex 15/16, 5 August 2016
- Erasmus Medical Center Rotterdam, The Netherland, #MEC-2016-036 - #NL56039.078.15, v.4, 7 March 2016.
- Hospital Clínico San Carlos, Madrid, Spain, # 15/532-E_BC, 16 September 2016
- Bellvitge University Hospital Barcellona, Spain, #PR204/15, 29 January 2016.
- Friedrich-Alexander University Erlangen-Nürnberg, Germany, #340_15B, 21 January 2016.
- Helsinki committee in Maccabi Healthcare services, Bait Ba-lev, Bat Yam, Israel, #45/2016, 24 July 2016.

## 3. Results

General characteristics of the study population are reported in Table 2. As may be seen from the Table, men and women were equally distributed in the SCOPE cohort at baseline (1256 women/1001 men) with a median age of 80.3 ± 4.1 years for women and 80.4 ± 4.1 years for men. Men differed from women with a significantly lower eGFR as determined by CKD-EPI, BIS and FAS equation (data see Table 2, significance for all equations applied *p* < 0.001), had a higher amount of muscle mass in average and performed significantly better in the SPPB (women SPPB 8.3 ± 3.1, men SPPB 9.3 ± 2.7, *p* < 0.001). Diabetes (*p* < 0.001), heart failure (*p* = 0.004), atrial fibrillation (*p* = 0.002) and myocardial infarction (*p* < 0.001) were more frequent in men than in women, arterial hypertension and stroke should a similar tendency without reaching the level of statistical significance.

**Figure 1:**
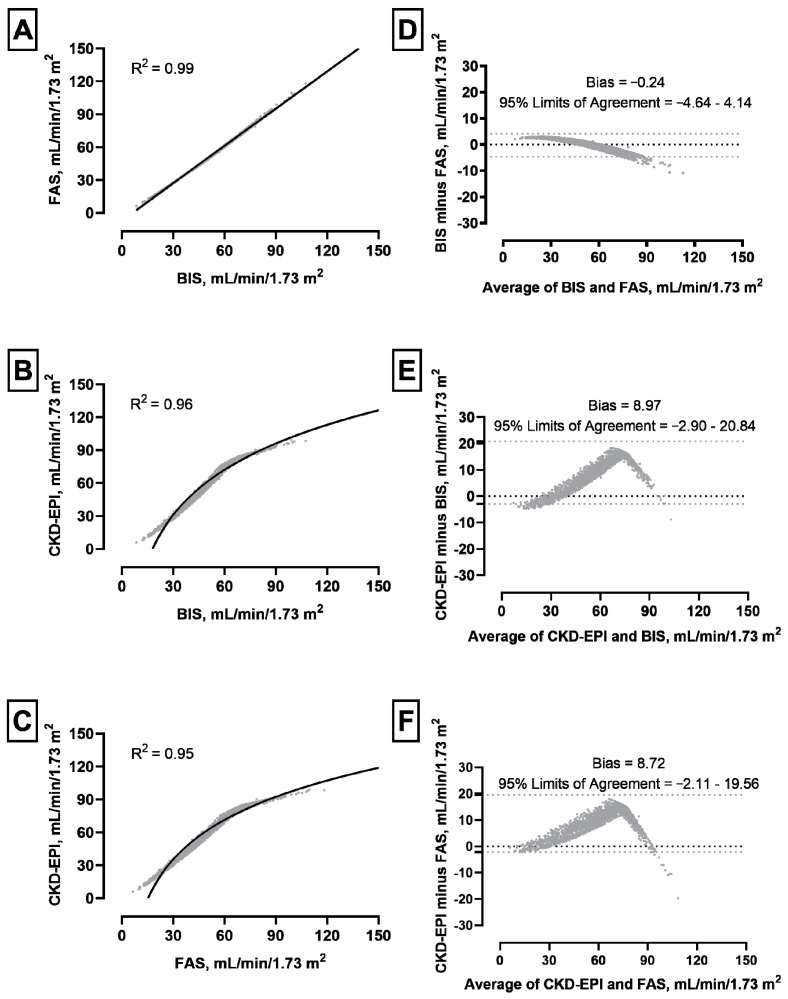
Crude correlations among eGFR equations (panels **A**,**B**,**C**) and Bland–Altman analysis (Panels **D**,**E**,**F**). Figure 1 shows the correlation of GFR values obtained when applying different equations for eGFR calculation. When testing CKD-EPI values towards the dynamic for BIS and FAS equations in the SCOPE cohort (Panels **E**,**F**), a relevant bias of 8.97 mL/min/1.73 m^2^ for the BIS equation and 8.72 mL/min/1.73 m^2^ for the FAS equation was detected, while the bias was much lower (−0.24 mL/min/1.73 m^2^). Negative values may be explained by the methodology chosen to plot the distribution of values.

When comparing levels of GFR calculated by CKD-EPI, BIS and FAS formula for the whole cohort of participants the average eGFR value was higher with CKD-EPI compared to BIS (*p* < 0.001) and FAS (*p* < 0.001) equations for the whole cohort (see Table 2 and Figure 1).

The three eGFR equations were strongly correlated each other, even if the correlations between CKD-EPI and BIS or FAS were less linear compared to that observed between BIS and FAS (Figure 1, panels A–C).

The Bland–Altman analysis showed that the bias between BIS and FAS was very small (−0.24 mL/min/1.73 m^2^); greater difference was observed only for patients with high eGFR values (Figure 1, panel D). In contrast, there was a significant difference in calculated GFR values between CKD-EPI and BIS and also between CKD-EPI and FAS (8.97 mL/min/1.73 m^2^ and 8.72 mL/min/1.73 m^2,^ respectively), peaking around 60 mL/min/1.73 m^2^ for both equations (CKD-EPI compared to BIS, CKD-EPI compared to FAS). Additionally, the 95% upper limits of agreement were 20.84 and 19.56 mL/min/1.73 m^2^, respectively (Figure 1, panels E and F).

Properties of eGFR equations to classify and stage CKD were significantly different (Table 3, Table 4 and Table 5). The prevalence of stage 2 was 59.1% according to CKD-EPI, 33.6% according to BIS and 34.3% according to FAS, while the corresponding figures for stage 3a and 3b were 19.2%, 38.9%, and 35.0%, and 12.0%, 20.0% and 19.9%, respectively (Table 2). Overall, κ value was 0.47 for both CKD-EPI vs. FAS and CKD-EPI vs. BIS, while the classificatory properties of BIS and FAS were found to be very similar (κ = 0.90) (Table 3). When applying the KDIGO stratification system to our study population, the prevalence of stage 2 was much more prevalent with CKD-EPI compared to FAS or BIS among patients without proteinuria or with moderate proteinuria, while differences among equations were smaller in patients with severe proteinuria (Table 3). It is worth noting that 574 (43%) out of 1335 patients classified in stage 2 by CKD-EPI were classified in stage 3a by FAS equation, and 216 out of 433 (50%) patients classified in stage 3a by CKD-EPI were classified in stage 3b by FAS equation. Similar findings were obtained when comparing CKD-EPI and BIS equation, while such a difference was negligible when comparing BIS and FAS equations (Table 4). Finally, disagreement between CKD-EPI and FAS or BIS was more evident among women (Table 5) than men (Table 6).

**Figure 2:**
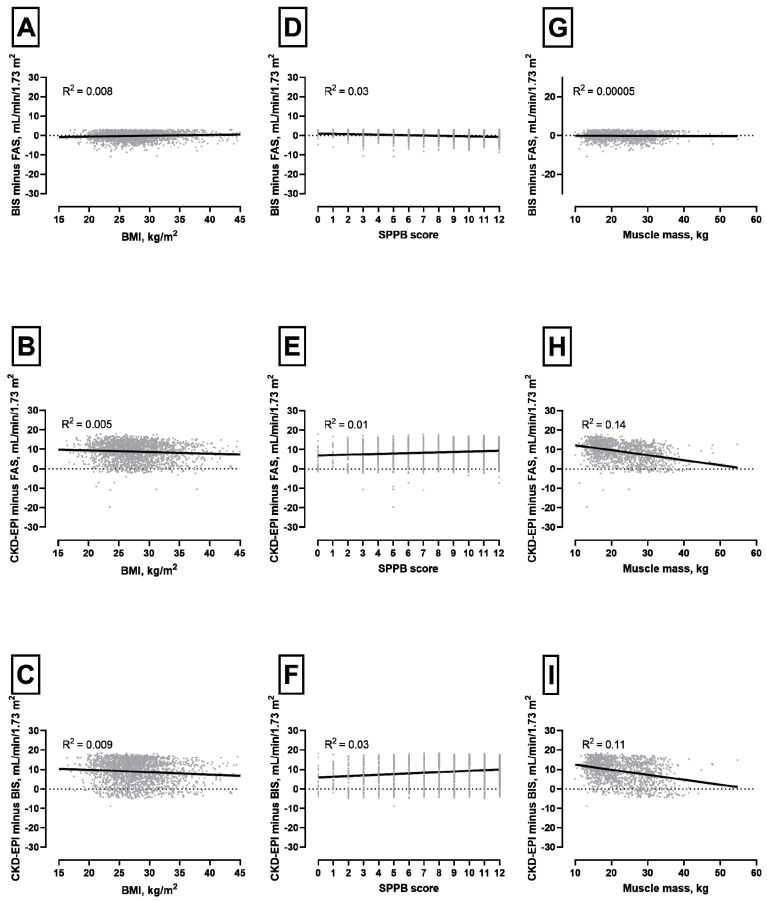
Graphical analysis of the impact of BMI (panels **A**–**C**), physical performance (Panels **D**–**F**) and muscle mass* (Panels **G**–**I**) on the difference among eGFR equations studied. *N = 1462. Figure 2 shows the graphic analysis referring to the point distribution of selected study variables in relation to the difference between two equations and the correlation of BMI, SPPB and muscle mass on different calculation models of glomerular filtration rate. The choice of design was adapted on the basis of the regression curve best fitting the distribution of values. As may be seen from the figure, there is no impact of BMI and physical performance quantified by SPPB in this model on sensitivity of BIS and FAS related calculation models as well as CKD-EPI including in the models. Muscle mass seems to have no impact in models including FAS and BIS (panel D), however, as soon as CKD-EPI is included muscle mass is negatively correlated to eGFR values (CKD-EPI and BIS; R^2^ = 0.14 panel H-CKD-EPI and BIS; R^2^ = 0.11 panel I).

BMI and SPPB score were not significantly correlated with difference among equations (Figure 2, panels A-F). The analysis regarding muscle mass was limited to 1462 patients undergoing BIA during the enrolment visit. The relationship between muscle mass and BIS minus FAS was not significant. Conversely, CKD-EPI minus FAS and CKD-EPI minus BIS increased together with decreasing muscle mass (Figure 2, panels G-I). Graphical analysis of the impact of serum creatinine (Panels A-C) and albumin-to-creatinine ratio (Panels D-F) on the difference among eGFR equations studied is shown in Figure S1.

## 4. Discussion

Overall, our findings clearly show that BIS and FAS equations may provide different estimates of GFR compared to CKD-EPI equation in a population of older outpatients. It is important to notice that the greatest difference is observed for eGFR values around 60 mL/min/1.73 m^2^. The fact that this range of GFR is of potential interest in daily clinical practice management of older subjects puts our results in the focus of experts as well as clinicians.

Determination of kidney function has been a topic of discussion among experts and may impact clinical management of patients as well as setting endpoints in future clinical studies [21]. Over the past decade, several attempts have been made to test creatinine-based mathematical models for determination of kidney function in different cohorts of patients and healthy subjects [14]. Furthermore, parameters like age, sex, muscle mass, may impact internal consistency of measurements of kidney function, especially in this cohort of older subjects [5]. It has been speculated that loss of muscle mass, which is common in the frail older persons, may impact production of creatine and ensuing low levels of serum creatinine even despite a depressed glomerular filtration rate (GFR) [6].

To date, only few studies including multimorbid older subjects, also including those with physical functional deficits, have focused on this burning issue for clinical practice, but also research. The older people participating in the SCOPE study represent a rich source and wealth in several dimensions: people older than 75 years and living in a community were recruited and followed up for two years on a voluntary basis in this observational study with a wide recruitment frame and only few exclusion criteria [20]. Not surprisingly, women are outnumbering men participants as it is a fact that women account for a higher percentage of older community dwelling persons in many EU countries nowadays [27]. The very open inclusion criteria concerning kidney function (only excluding people with an initial eGFR < 15 mL/min^−1^ during recruitment phase) furthermore contributed to a real-life picture when treating older people in clinical practice in the EU.

Given the fact that recent data on prevalence of CKD clearly demonstrated a sex bias for CKD, men being at higher risk for development of CKD [28], it was the major interest of the study team to also address sex differences when looking at the impact of the method used to calculate eGFR on staging of CKD based on creatinine- and albuminuria-based guidelines [4]. The access to data from a cohort older than 75 years, many of them multimorbid and prone to loss of muscle mass, made it possible for the consortium to further test the hypothesis that impaired physical performance and loss of muscle mass may impact the degree of agreement among eGFR equations in this cohort of older European citizens [20].

Several studies reported on discrepancies between eGFR values obtained with different equations, but only a few of them included the most recently published BIS and FAS equations at the same time. A former study showed that the average difference between creatinine-based CKD-EPI and BIS was 9.5 mL/min/1.73 m^2^ in a population of 828 community-dwelling older people [16], which is very close to the 8.97 mL/min/1.73 m^2^ difference observed in the present study. The average 8.72 mL/min/1.73 m^2^ difference between CKD-EPI and FAS, as well as the negligible average difference between BIS and FAS are not surprising given the fact that FAS has been designed to match the BIS equation for ages >70 years [11]. Additionally, FAS was recently reported to predict eGFR calculated by the creatinine/cystatin C-based CKD-EPI equation with a median bias 10.2 mL/min/1.73 m^2^ (95%CI = 9.2–10.9) in a population of 1913 Chinese CKD patients aged 50.3 ± 18.2 years [29]. Interestingly, the above differences were observed despite the good average diagnostic performance of CKD-EPI, BIS and FAS equations in predicting measured GFR. Indeed, da Silva Selistre recently reported that the median difference between CKD-EPI and FAS in predicting measured GFR was −2.0 (95%CI = −3.5; −2.5) mL/min/1.73 m^2^, and the corresponding figure for the difference between CKD-EPI and BIS was 0.0 (95%CI = −1.5; 0.5). Thus, if we consider such a small difference between equations with respect to gold-standard measured GFR, the average differences between CKD-EPI and BIS or FAS equations observed in our study would be unexpected.

Nevertheless, eGFR equations are known to work well in populations for which they had been developed [30]. The FAS equation was originally developed in a life-span perspective to allow eGFR calculation from childhood to older age [11], while the BIS equation was specifically developed in a population of people aged 70 years or more [9]. At variance, CKD-EPI was developed in a pooled population with a wide age range (50 ± 15 years), but only 13.0% of the development and validation population was aged 65 years or more [8]. On the other hand, our study confirms the good agreement between BIS and FAS equations, which, given their history and intention of development, is less surprising.

As regards potential sources of discrepancy among eGFR equations, serum creatinine, and muscle mass are main correlates also impacting known sex differences of the CKD-EPI and BIS or FAS equations. Finally, differences among eGFR equations are much more evident among women and significantly impact the KDIGO staging of CKD. It is therefore evident that choosing older people to undergo CKD-related diagnostic procedures and/or treatments will change depending on which equation is used to assess eGFR.

In our analysis, we tried to estimate the agreement in terms of CKD staging between equations, i.e., from the practitioner’s perspective. Indeed, “misclassification” has important clinical implications in terms of staging and management of CKD, especially between stage 2 and 3 CKD. Given the high prevalence of stage 3 CKD and the highly variable risk of mortality as well as other negative outcomes in this group, current guidelines include a distinction between CKD stage 3a and 3b [4]. The risk associated with stage 3a CKD in older patients is still under discussion [31], and GFR level at which the risk of mortality starts to increase may be lower among older patients compared to younger ones [32]. Additionally, older people with eGFR<60 mL/min/1.73 m^2^ exhibit a slow progression of CKD [33]. However, current guidelines do not calibrate the definition of CKD for age and suggest many stage-specific therapeutic measures [4]. Relative “misclassification” is also more common in women in our study. Sex represents a relevant non-GFR determinant of serum creatinine [34], and clinically relevant discrepancies between eGFR equations were found to be more frequent among women aged 75 or more [35].

Failure to correctly classify older CKD patients also poses significant challenges in managing kidney cleared medications, especially among older patients with multiple chronic diseases treated with polypharmacy regimens. As an example, guideline recommendations suggest careful dosing of several hypoglycemic agents in patients with eGFR <60 mL/min/1.73 m^2^ [36]. Finally, disagreement between equations may have important implications when prescribing or dosing several other drugs [37], including direct oral anticoagulants [38], in terms of missing contraindication or dose reduction recommendation on one side, and underuse or underdosing on the other side.

Additionally, the difference between CKD-EPI and BIS or FAS equations increased together with reducing ACR and muscle mass in our study. The greatest difference between CKD-EPI and BIS or FAS equations was observed for lower ACR and muscle mass values. Creatinine is a metabolic product of creatine and phosphocreatine arising from the muscle compartment which is directly related to muscle mass [39], and albuminuria also is known to be associated with muscle mass. Sarcopenia is a common occurrence among older adults and its prevalence increases dramatically with decreasing kidney function. In CKD patients, the prevalence of sarcopenia was found to be approximately 40% for eGFR = 60–89 mL/min/1.73 m^2^, and approximately 60% for eGFR < 60 mL/min/1.73 m^2^ [40]. Thus, the above findings together with the observation that the difference between BIS and FAS was only marginally affected by serum creatinine, ACR and muscle mass in the present study, suggest that the population of the SCOPE study may be on one side very similar to that used for the development of the BIS and FAS equations (i.e., an older population aged 75 or more that includes sarcopenic patients), and on the other side very different from that used for the development of the CKD-EPI equation (i.e., a younger population including only 13% of people aged 65 or more). Finally, part of the sex differences observed in our study for the different GFR equations may be attributed to the lower muscle mass of women participating in the SCOPE study (Table 1) [41].

## 5. Limitations and Strengths

Some limitations of the present study deserve consideration. Our study did not include a direct measurement of GFR. Thus, we cannot draw any definitive conclusion about the diagnostic accuracy of the study equations against a gold standard assessment. Indeed, experimental evidence suggests that intravenous inulin and iohexol may partially undergo extrarenal clearance [42,43], which may represent a not negligible potential source of error when measuring GFR for developing or validating eGFR equations. Nevertheless, the amount of extrarenal clearance of GFR markers in humans is unknown and is worthy of future investigations. Additionally, patients with eGFR < 15 mL/min/1.73 m^2^ were excluded. Nevertheless, this limitation is likely to have a minor role in the present study given the good agreement observed among equations in patients with severe CKD.

This study also has important strengths, including the enrollment of a real-world population of older outpatients and the opportunity to investigate the impact of objectively measured physical performance and body composition on the observed difference among equations.

## 6. Conclusions

Our results show that CKD-EPI and BIS or FAS equations cannot be considered interchangeable to assess eGFR in a population of older outpatients. Indeed, despite a fair overall concordance, the respective eGFRs differ significantly in the range of GFR corresponding to CKD stages 2–3b, and this could have a dramatic impact on our diagnostic and therapeutic approach. While our study does not allow to draw a definitive conclusion on diagnostic accuracy of each individual equation, BIS and FAS equations provided very similar eGFR values and the difference between BIS and FAS seems to be unaffected by muscle mass. At variance, muscle mass seems to represent a major source of discrepancy between CKD-EPI and BIS or FAS. Thus, our findings suggest that the two most recent BIS and FAS equations specifically developed in older patients may be very useful for clinical assessment of eGFR in a population of older outpatients aged >75 years. Additionally, their substantial overlap would minimize discrepancy issues when monitoring progression of CKD. Finally, the impact of muscle mass on CKD staging and its predictivity, as well as the clinical usefulness of muscle mass assessment to decide which equation to use in clinical practice deserve to be further investigated.

## Figures and Tables

**Table 1:** Estimated glomerular filtration rate equations used in the present study.

Reference Study	Equation	
CKD-EPI [8]	Women (Scr ≤ 0.7) (Scr > 0.7)	eGFR = 144 × (Scr/0.7)^−0.329^ × (0.993)^Age^ eGFR = 144 × (Scr/0.7)^−1.209^ × (0.993)^Age^
	Men (Scr ≤ 0.9) (Scr > 0.9)	eGFR = 141 × (Scr/0.9)^−0.411^ × (0.993)^Age^ eGFR = 141 × (Scr/0.9)^−1.209^ × (0.993)^Age^
BIS [9]	3736 × creatinine^−^^0.87^ × age^−^^0.95^ × 0.82 (if women)	
FAS [11]	(107.3/(creatinine/Q)) × 0.988^(Age-40)^ for age >40 years Q = median Scr value for age-/sex-specific healthy populations	

CKD-EPI, Chronic kidney disease–Epidemiologic Collaboration; BIS, Berlin Initiative Study; FAS, Full Age Spectrum.

**Table 2:** General characteristics of the study population in the SCOPE project.

		All Patients N = 2257	Women N = 1256	Men N = 1001	*p*-Value
Age, years		80.3 ± 4.1	80.3 ± 4.1	80.4 ± 4.1	0.671
Sex, women		1256 (55.6)	-	-	-
Body mass index, kg/m^2^		27.8 ± 4.7	27.9 ± 4.9	27.6 ± 4.5	0.153
Serum creatinine, mg/dL		1.11 ± 0.56	0.93 ± 0.41	1.33 ± 0.64	<0.001
CKD-EPI eGFR, mL/min/1.73 m^2^		63.8 ± 19.4	65.4 ± 18.1	58.9 ± 20.5	<0.001
	90 or more	43 (1.9)	32 (2.5)	11 (1.1)	
	60–90	1335 (59.1)	807 (64.3)	528 (52.7)	
	45–60	433 (19.2)	240 (19.1)	193 (19.3)	
	30–45	271 (12.0)	112 (8.9)	159 (15.9)	
	<30	175 (7.8)	65 (5.2)	110 (11.0)	
BIS eGFR, mL/min/1.73 m^2^		54.6 ± 15.2	55.5 ± 14.8	51.1 ± 14.9	<0.001
	90 or more	9 (0.4)	7 (0.6)	2 (0.2)	
	60–90	759 (33.6)	471 (37.5)	288 (28.8)	
	45–60	877 (38.9)	499 (39.7)	378 (37.8)	
	30–45	451 (20.0)	213 (17.0)	238 (23.8)	
	<30	161 (7.1)	66 (5.3)	95 (9.5)	
FAS eGFR, mL/min/1.73 m^2^		55.0 ± 17.3	55.4 ± 16.9	51.7 ± 17.0	<0.001
	90 or more	29 (1.3)	18 (1.4)	11 (1.1)	
	60–90	775 (34.3)	467 (37.2)	308 (30.8)	
	45–60	791 (35.0)	454 (36.1)	337 (33.7)	
	30–45	450 (19.9)	227 (18.1)	223 (22.3)	
	<30	212 (9.4)	90 (7.2)	122 (12.2)	
ACR, mg/g		100 ± 480	77.1 ± 390	177 ± 599	<0.001
	<30	1648 (73.0)	992 (79.0)	656 (65.5)	
	30–300	458 (20.3)	216 (17.2)	242 (24.2)	
	>300	151 (6.7)	48 (3.8)	103 (10.3)	
Muscle mass, kg (N = 1462)		22.7 ± 6.8	18.0 ± 3.8	29.0 ± 4.4	<0.001
Short Physical Performance Battery score		8.7 ± 2.9	8.3 ± 3.1	9.3 ± 2.7	<0.001
Hypertension		1734 (76.8)	972 (76.6)	772 (77.1)	0.767
Diabetes Mellitus		569 (25.2)	264 (21.0)	305 (30.5)	<0.001
Heart Failure		373 (16.5)	182 (14.5)	191 (19.1)	0.004
Atrial fibrillation		344 (15.2)	165 (1.1)	179 (17.9)	0.002
Myocardial infarction		217 (9.6)	75 (6.0)	142 (14.2)	<0.001
Stroke		131 (5.8)	61 (4.9)	70 (7.0)	0.031

Table 2 shows baseline characteristics of the participants recruited to the SCOPE study. As may be seen from the Table, women and men were equally distributed in the cohort, ranging at a mean age of 80.4 ± 4.1 years for women and 80.3 ± 4.1 years for men. Diabetes, atrial fibrillation, heart failure and myocardial infarction were more frequent in men than in women. So were hypertension and stroke, however, not reaching the level of statistical significance. Men performed better in SPPB and had greater muscle mass than women. According to data outlined, men had lower glomerular filtration rates, whatever was the equation used to calculate eGFR and also had higher loss of albumin in urine.

**Table 3:** Prevalence of KDIGO stages with three different equations stratified by sex.

	All Patients, N = 2257			Men, N = 1001			Women, N = 1256		
CKD-EPI	ACR (mg/g)			ACR (mg/g)			ACR (mg/g)		
	<30	30–300	>300	<30	30–300	>300	<30	30–300	>300
G1 (90 or more)	35 2.1%	7 1.5%	1 0.7%	8 1.2%	3 1.3%	0	27 2.7%	4 1.8%	1 2.1%
G2 (60-90)	1133 68.8%	188 41.3%	13 8.6%	433 65.9%	86 36.1%	9 8.7%	700 70.7%	102 47.0%	4 8.3%
G3A (45-60)	311 18.9%	98 21.5%	24 15.8%	128 19.5%	50 21.0%	15 14.4%	183 18.5%	48 22.1%	9 18.8%
G3B (30-45)	133 8.1%	84 18.5%	51 33.6%	69 10.5%	52 21.8%	36 34.6%	64 6.5%	32 14.7%	15 31.3%
G4-5 (<30)	35 2.1%	78 17.1%	63 41.4%	19 2.9%	47 19.7%	44 42.3%	19 2.9%	31 14.3%	19 39.6%
**BIS**									
G1 (90 or more)	6 0.3%	3 0.8%	0	1 0.2%	1 0.4%	0	5 0.5%	2 0.9%	0
G2 (60-90)	655 39.8%	97 21.3%	7 4.6%	247 37.6%	37 15.5%	5 4.8%	408 41.3%	60 27.6%	2 4.2%
G3A (45-60)	710 43.1%	143 31.4%	23 15.1%	285 43.4%	80 33.6%	12 11.5%	425 42.9%	63 29.0%	11 22.9%
G3B (30-45)	244 14.8%	140 30.8%	64 42.1%	109 16.6%	79 33.2%	48 46.2%	135 13.6%	61 28.1%	16 33.3%
G4-5 (<30)	32 1.9%	72 15.8%	58 38.2%	15 2.3%	41 17.2%	39 37.5%	17 1.7%	31 14.3%	19 39.6%
**FAS**									
G1 (90 or more)	22 1.3%	6 1.3%	1 0.7%	8 1.2%	3 1.3%	0	14 1.4%	3 1.4%	1 2.1%
G2 (60-90)	669 40.7%	101 22.2%	6 3.9%	263 40.0%	42 17.6%	5 4.8%	406 41.1%	59 27.2%	1 2.1%
G3A (45-60)	641 38.9%	131 28.8%	17 11.2%	254 38.7%	72 30.3%	9 8.7%	387 39.1%	59 27.2%	8 16.7%
G3B (30-45)	267 16.2%	127 27.9%	53 34.9%	109 16.6%	72 30.3%	40 30.5%	158 16.0%	55 25.3%	13 27.1%
G4-5 (<30)	48 2.9%	90 19.8%	75 49.3%	23 3.5%	49 20.6	50 48.1%	25 2.5%	41 18.9%	25 52.1%

Table 3 shows the staging of SCOPE participants (also stratified by sex) using different equations to calculate creatinine-based eGFR by ACR values. Colour coding was used according to KDIGO: Green = low risk, Yellow = moderately increased risk, Orange = high risk, Red = very high risk (4). As may be seen, there is a significant shift of participants from stage 2 of CKD when staged according to CKD-EPI formula compared to BIS and FAS formula to stage 3a and from 3a to 3b. This is an important finding as clinical management of patients is highly impacted by stage of CKD and glomerular filtration rate (4).

**Table 4:** Agreement among eGFR equations studied for the whole group of participants of the SCOPE study.

		**CKD-EPI**						
		**90 OR MORE (N = 43)**	**90-60 (N = 1335)**	**60-45 (N = 433)**	**45-30 (N = 271)**	**<30 (N = 175)**	**κ**	***p***
							0.47	0.001
FAS	90 OR MORE	29						
		67.4%						
	90-60	14	761					
		32.6%	57.0%					
	60-45		574	217				
			43.0%	50.1%				
	45-30			216	234			
				49.9%	86.3%			
	<30				37	175		
					13.7%	100.0%		
							0.47	0.001
BIS	90 OR MORE	9						
		20.9%						
	90-60	34	725					
		79.1%	54.3%					
	60-45		610	267				
			45.7%	61.7%				
	45-30			166	267	18		
				38.3%	98.5%	10.3%		
	<30				4	157		
					1.5%	89.7%		
		**BIS**						
		**90 OR MORE (N = 9)**	**90-60** **(N = 759)**	**60-45** **(N = 877)**	**45-30** **(N = 451)**	**<30** **(N = 161)**	**κ**	***p***
							0.90	0.001
FAS	90 OR MORE	9	20					
		100.0%	2.6%					
	90-60		738	37				
			97.2%	4.2%				
	60-45		1	790				
			0.1%	90.1%				
	45-30			50	400			
				5.7%	88.7%			
	<30				51	161		
					11.3%	100.0%		

Table 4 shows the inter-relationship between CKD-EPI, BIS and FAS equations for the different stages of CKD according to KDIGO Guidelines. As may be seen for values obtained for the whole cohort of participants, Cohen’s Kappa was high for the relation between BIS and FAS equation values, but rather fair when relating CKD-EPI to BIS (κ = 0.47) and FAS equation results (κ = 0.47).

**Table 5:** Agreement among eGFR equations studied in women comparing CKD-EPI, BIS and FAS formula.

		**CKD-EPI**						
		**90 OR MORE (N = 32)**	**90-60 (N = 807)**	**60-45 (N = 240)**	**45-30 (N = 112)**	**<30 (N = 65)**	κ	***p***
							0.36	0.001
FAS	90 OR MORE	18						
		56.3%						
	90-60	14	453					
		43.8%	56.1%					
	60-45		354	100				
			43.9%	41.7%				
	45-30			140	87			
				58.3%	77.7%			
	<30				25	65		
					22.3%	100.0%		
							0.41	0.001
BIS	90 OR MORE	7						
		21.9%						
	90-60	25	446					
		78.1%	55.3%					
	60-45		361	138				
			44.7%	57.5%				
	45-30			102	109	2		
				42.5%	97.3%	3.1%		
	<30				3	63		
					2.7%	96.9%		
		**BIS**						
		**90 OR MORE (N = 7)**	**90-60** **(N = 471)**	**60-45** **(N = 499)**	**45-30** **(N = 213)**	**<30** **(N = 66)**	**κ**	***p***
							0.90	0.001
FAS	90 OR MORE	7	11					
		100.0%	2.3%					
	90-60		459	8				
			97.5%	1.6%				
	60-45		1	453				
			0.2%	90.8%				
	45-30			38	188			
				7.6%	88.7%			
	<30				24	66		
					11.3%	100.0%		

Table 5 shows the inter-relationship between CKD-EPI, BIS and FAS equations for the different stages of CKD according to KDIGO Guidelines for women participants in the SCOPE study. As may be seen for values obtained for women, Cohen’s Kappa was high for the relation between BIS and FAS equation values (κ = 0.90), but even more fair when relating CKD-EPI to BIS (κ = 0.41) and FAS equation results (κ = 0.36) compared to men in this study.

**Table 6:** Agreement among eGFR equations studied in men comparing CKD-EPI, BIS and FAS formula.

		**CKD-EPI**						
		**90 OR MORE (N = 11)**	**90-60 (N = 528)**	**60-45 (N = 193)**	**45-30 (N = 159)**	**<30 (N = 110)**	κ	***p***
							0.57	0.001
FAS	90 OR MORE	11						
		100%						
	90-60		308					
			58.3%					
	60-45		220	117				
			41.7%	60.6%				
	45-30			76	147			
				39.4%	92.5%			
	<30				12	110		
					7.5%	100.0%		
							0.53	0.001
BIS	90 OR MORE	2						
		18.2%						
	90-60	9	279					
		81.8%	52.8%					
	60-45		249	129				
			47.2%	66.8%				
	45-30			64	158	16		
				33.2%	99.4%	14.5%		
	<30				1	94		
					0.6%	85.5%		
		**BIS**						
		**90 OR MORE (N = 2)**	**90-60 (N = 288)**	**60-45 (N = 378)**	**45-30 (N = 238)**	**<30 (N = 95)**	**κ**	***p***
							0.89	0.001
FAS	90 OR MORE	2	9					
		100.0%	3.1%					
	90-60		279	29				
			96.9%	7.7%				
	60-45			337				
				89.2%				
	45-30			12	211			
				3.2%	88.7%			
	<30				27	95		
					11.3%	100.0%		

Table 6 shows the inter-relationship between CKD-EPI, BIS and FAS equations for the different stages of CKD according to KDIGO Guidelines for men participants in the SCOPE study. As may be seen for values obtained for men, Cohen’s Kappa was high for the relation between BIS and FAS equation values (κ = 0.89), but rather fair when relating CKD-EPI to BIS (κ = 0.53) and FAS equation results (κ = 0.57).

## Supplementary Materials

The following are available online at https://www.mdpi.com/2077-0383/9/2/294/s1, Figure S1: Impact of serum creatinine (Panels A-C) and albumin-to-creatinine ratio (Panels D-F) on the difference among eGFR equations studied.
