# Supplementary figures and images for: Clinical Implications of Estimating Glomerular Filtration Rate with Three Different Equations among Older People. Preliminary Results of the Project “Screening for Chronic Kidney Disease among Older People across Europe (SCOPE)”
Source: J Clin Med. 2020 Jan 21;9(2):294. doi: 10.3390/jcm9020294 (PMC7074235; doi:10.3390/jcm9020294)

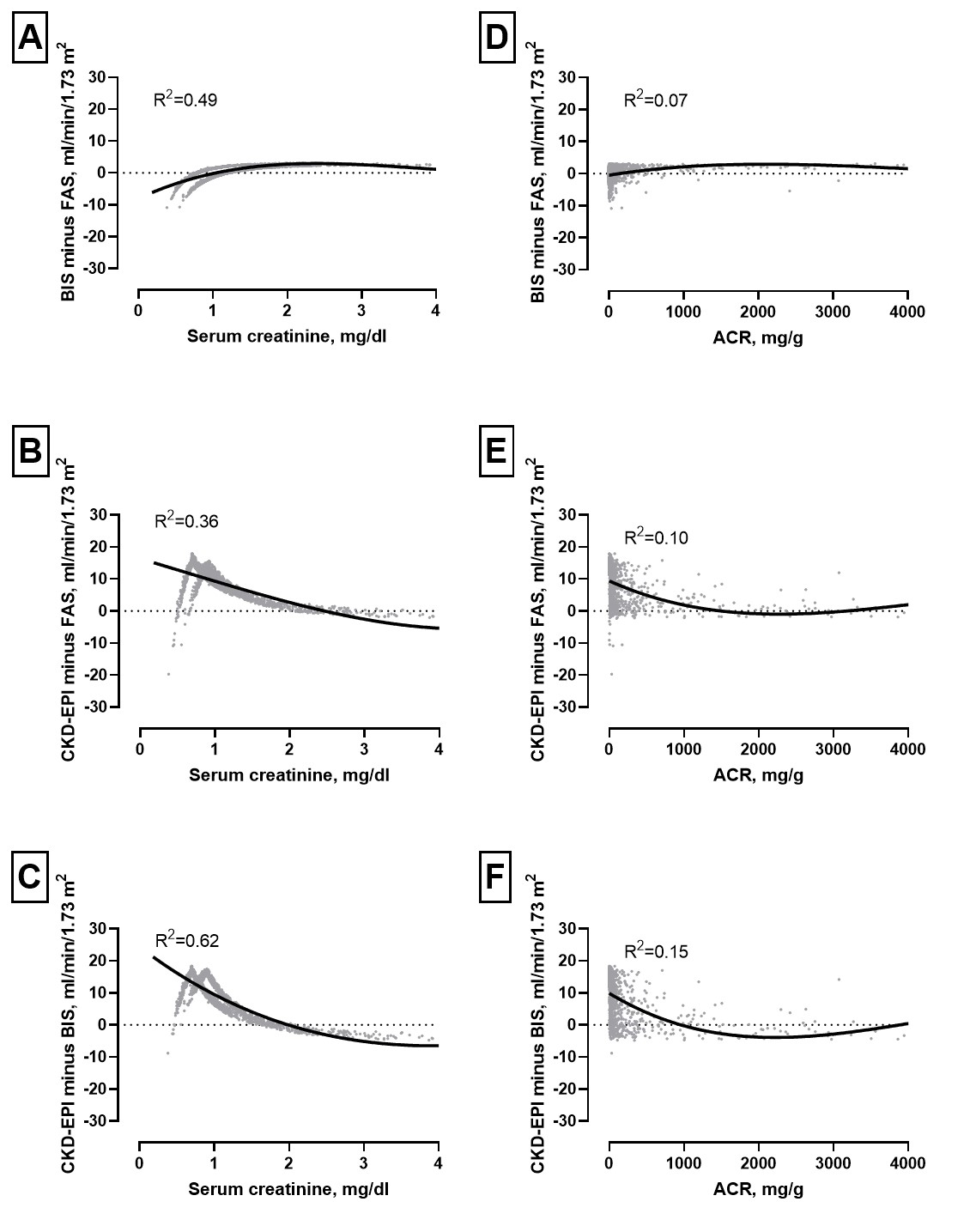

Supplement: Supplementary file 1 [file jcm-09-00294-s001.zip › Figure_S1.jpg]
